# Acute Pancreatitis After Intraoperative Cholangiogram in a Patient With Obstructive Jaundice: A Case Report

**DOI:** 10.7759/cureus.35743

**Published:** 2023-03-03

**Authors:** Katrina Ng, Sabu Thomas

**Affiliations:** 1 General Surgery, Kalgoorlie Health Service, Kalgoorlie, AUS

**Keywords:** cholelithiasis, intra-operative cholangiogram, acute pancreatitis, laporoscopic cholecystectomy, choledocholithiasis

## Abstract

Laparoscopic cholecystectomy with intraoperative cholangiogram is commonly performed, especially when there is suspicion of choledocholithiasis. We present a case of acute pancreatitis post-procedure for management of acute cholecystitis and suspicion of distal common bile duct sludge, potentially caused by the dislodgement of microlithiasis, which resolved on its own with supportive management. This case emphasizes the need to consider acute pancreatitis as a rare but possible post-cholecystectomy complication in a patient who continues to be unwell post-operatively.

## Introduction

Acute calculus cholecystitis has a high incidence in the general population. The prevalence of gallstones in the general population is 10-15% [1], with some differences across countries, where the annual incidence of this population suffering from acute cholecystitis is 0.3% [2]. The standard of care recommended by the World Society of Emergency Surgery (WSES) is early laparoscopic cholecystectomy where possible [1]. In the case of high suspicion for common bile duct (CBD) stones, further investigation with preoperative endoscopic retrograde cholangiopancreatography (ERCP), intraoperative cholangiogram (IOC), or laparoscopic ultrasound (LUS) should be performed. IOC has the benefit of avoiding the complications of ERCP, including pancreatitis, cholangitis, bleeding, and duodenal perforation. A large study with more than 11,000 patients undergoing ERCP found the pancreatitis risk to be 2.6% [3]. We present a case of pancreatitis post-laparoscopic cholecystectomy and IOC.

## Case presentation

**Figure 1:**
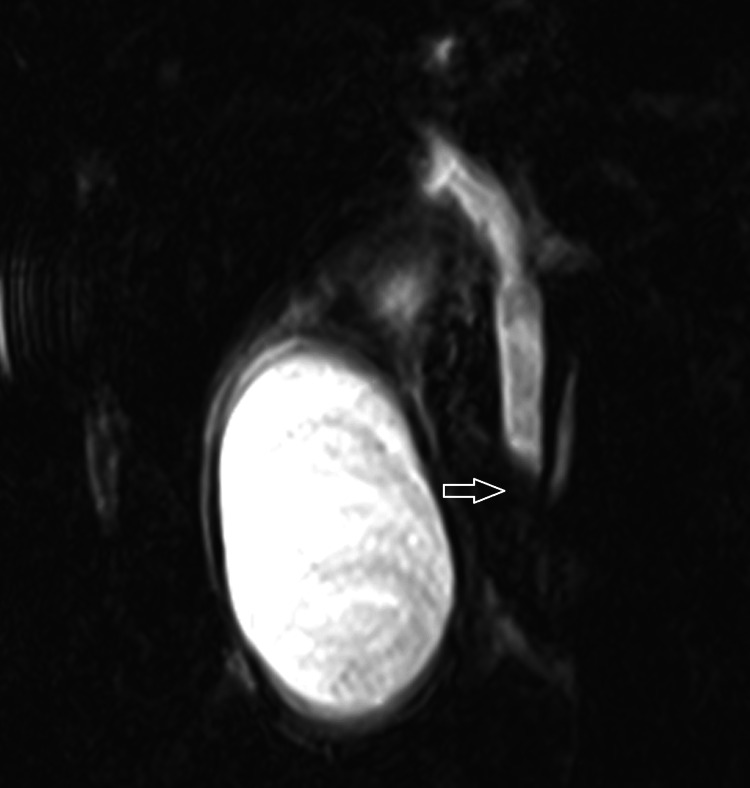
MRCP showing possible sludge in distal CBD (white arrow). MRCP: magnetic resonance cholangiopancreatography, CBD: common bile duct.

A 37-year-old female presented to our hospital’s emergency department with a two-day history of epigastric pain and vomiting. She had an abdominal ultrasound scan a year prior to her current presentation, which showed multiple small calculi in her gallbladder without evidence of cholecystitis. Common bile duct (CBD) was 8 mm. Her liver function tests (LFTs) were normal then. She has a history of sleeve gastrectomy and, later, a mini gastric bypass three years ago for weight loss. Her observations were normal; she had right upper quadrant tenderness and a positive Murphy’s sign on examination. Laboratory examination showed deranged LFTs with a total bilirubin of 20 µmol/L, alanine aminotransferase (ALT) of 168 U/L, alkaline phosphatase (ALP) of 222 U/L, and gamma-glutamyl transferase of 166 U/L. The white blood cell count was raised to 11.38 × 10^9^/L, with neutrophilia of 8.23 × 10^9^/L. C-reactive protein (CRP) was high at 145 mg/L. Serum lipase was normal at 139 (reference range: 20-210). She was started on intravenous ceftriaxone and metronidazole and was further investigated with another abdominal ultrasound on the day of admission. It showed a thickened gallbladder wall of 5 mm with pericholecystic fluid, but no gallstones were seen. CBD measured 7 mm, similar to the previous. The following day, total bilirubin climbed to 45 µmol/L, and therefore, magnetic resonance cholangiopancreatography (MRCP) was performed, which again demonstrated a thickened gallbladder wall with pericholecystic fluid and was also suspicious for biliary sludge in the distal CBD (Figure 1). Again, no calculi in the gallbladder were appreciated. The pancreas was normal.

**Figure 2:**
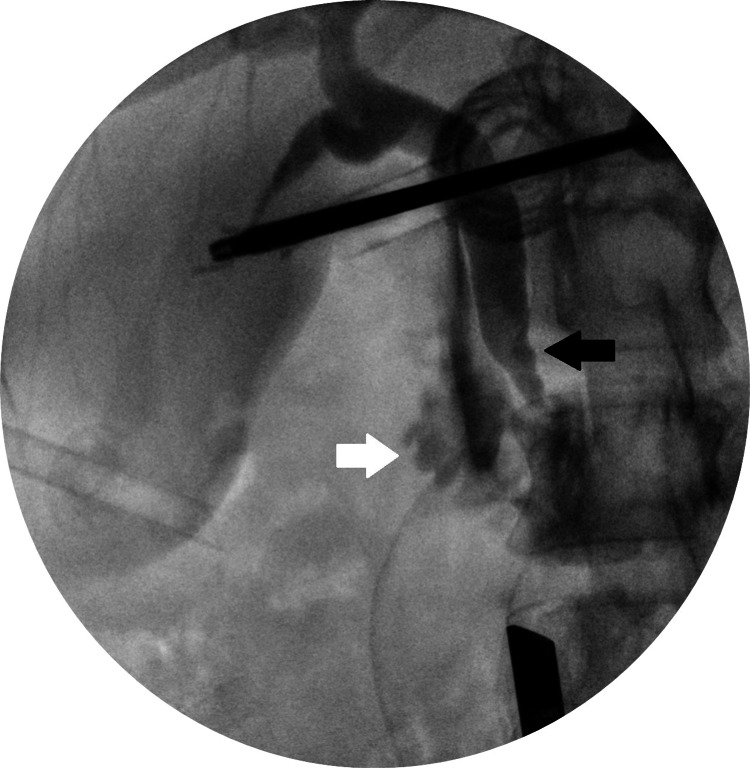
Intraoperative cholangiogram showing free flow into the duodenum (white arrow) and no filling defects in CBD (black arrow). CBD: common bile duct.

As she had cholecystitis, she then underwent laparoscopic cholecystectomy a few hours after MRCP, with a plan for trans-cystic bile duct exploration. However, the intraoperative cholangiogram showed free flow into the duodenum without any filling defects (Figure 2), and therefore, no bile duct exploration was performed. The following day she spiked a fever, had ongoing epigastric pain, and her CRP remained raised at 160 mg/L. Her bilirubin was still elevated at 41 µmol/L. Serum lipase was done two days post-procedure and was raised at 899, confirming the diagnosis of acute pancreatitis. She was discharged on the third day once she had clinically improved. LFTs taken one-week post-surgery showed improvement of total bilirubin to 18 (reference range <20), which is within normal limits. Her pain had also improved, and she made an uneventful recovery.

## Discussion

Intraoperative cholangiogram (IOC) is commonly performed during cholecystectomy, especially when there’s a moderate-to-high risk of a common bile duct stone. It involves inserting a 4-6 Fr catheter (Cook Medical, Bloomington, IN) into the cystic duct and injecting contrast; at our center, we use Urografin® (Bayer, Germany) through a 20-ml syringe flushed manually. While it is a common procedure often performed along with laparoscopic cholecystectomy, there have been no instances of pancreatitis as a complication reported in the literature. In a retrospective study by Morgan et al. with 435 patients who underwent IOC, three patients developed pancreatitis, two who underwent ERCP for choledocholithiasis, which pancreatitis was attributed to, and one who had hyperamylasemia seven days post-operatively and was diagnosed with papillary stenosis and microlithiasis during ERCP. The study concluded that IOC was not associated with pancreatitis [4]. No other articles investigating an association between IOC and pancreatitis were found.

Sphincter of Oddi stenosis (SOS) is defined by an abnormal narrowing caused by inflammation or scarring, such as pancreatitis, passage of gallstones, iatrogenic injury, infection, and adenomyosis. It is diagnosed with an elevated basal pressure of >40 mm Hg. SOS has been implicated in recurrent acute idiopathic pancreatitis in up to 20% of patients [5]. While this patient has likely passed gallstones in the past, as evidenced by stones no longer present compared to a year ago and a mildly dilated bile duct, the free flow of contrast into the duodenum during a cholangiogram makes her less likely to have SOS.

Another potential cause of acute pancreatitis is microlithiasis. In patients with recurrent pancreatitis without an obvious cause, microlithiasis is found in 13-70% of patients through bile salt analysis or endoscopic ultrasound (EUS) [5-8]. It is thought that microlithiasis might cause functional obstruction of the sphincter of Oddi, causing papillitis or papillary spasm. Recurrent passage of calculi can also lead to papillary stenosis [9]. Our patient was suspected to have biliary sludge in the distal CBD at the time of the MRCP but without evidence of pancreatitis. She subsequently developed acute pancreatitis, diagnosed two days after the cholecystectomy. While unable to be proven, sludge lying in the distal CBD might have been disturbed and dislodged during injection of contrast through the cystic duct, as it is in close proximity to the pancreatic duct, sludge might have refluxed into the pancreatic duct during injection of contrast through the cystic duct, causing pancreatic duct injury. Free flow of contrast into the duodenum makes stenosis or papillary dysfunction less likely. Subsequently, raised bilirubin could be secondary to biliary obstruction from pancreatitis, where the pancreas was swollen around the duct, causing extrinsic compression. Alternatively, the passage of sludge during IOC might have led to papillitis and subsequently acute pancreatitis. Given she had no evidence of pancreatitis based on normal lipase and normal pancreas on MRCP up to a few hours before the procedure, the presence of biliary sludge in CBD at that stage did not lead to pancreatitis.

## Conclusions

We report a case of acute pancreatitis post-intra-operative cholangiogram on a background of sludge in the distal common bile duct. While it is unlikely that the cholangiogram on its own caused pancreatitis, surgeons should keep into consideration pancreatitis as a diagnosis in a patient who continues to be unwell after what seemed like a definitive intervention, where interrogation of the biliary tract is involved, especially when there is a history of common bile duct calculi or sludge. It could possibly be caused by papillitis from the passage of calculi or sludge, or turbulence during the flushing of contrast leading to the reflux of sludge into the pancreatic duct.
